# Transfemoral Transcatheter Aortic Valve Implantation for Severe Aortic Stenosis in a Patient With Prior Tendyne Transcatheter Mitral Valve Replacement: Expanding the Boundaries of Transcatheter Valve Therapy

**DOI:** 10.1016/j.shj.2026.100802

**Published:** 2026-01-23

**Authors:** Rajan Rehan, Ben McDonough, Bernard Prendergast, Simon Redwood

**Affiliations:** Cardiovascular Department, Heart Lung Critical Care Group, Guy’s and St Thomas’ NHS Foundation Trust, London, UK; Department of Cardiovascular Medicine, School of Cardiovascular and Metabolic Medicine & Sciences, King’s College London, London, United Kingdom

## Abstract

• Transfemoral transcatheter aortic valve implantation can be safely performed in patients with prior Tendyne transcatheter mitral valve replacement.
• Careful preprocedural computed tomography planning allows assessment of left ventricular outflow tract obstruction risk and spatial interaction between the Tendyne frame and the aortic annulus.
• Use of a self-expanding transcatheter aortic valve may minimize the risk of device interaction in this complex anatomical setting.

With the growing adoption of transcatheter mitral valve replacement (TMVR), the *Tendyne system* (Abbott, Santa Clara, CA) represents the largest cohort of patients, supported by the longest available follow-up data.^1^ The Tendyne bioprosthesis incorporates a self-expanding D-shaped nitinol frame designed to conform to the native mitral annulus. This design may alter the left ventricular geometry and narrow the left ventricular outflow tract (LVOT), making subsequent transcatheter procedures, particularly transcatheter aortic valve implantation (TAVI), anatomically and technically challenging.

We report the case of a 78-year-old man with a prior TMVR using a Tendyne bioprosthesis 3 years earlier for degenerative mitral regurgitation, who presented with progressive congestive cardiac failure (New York Heart Association class III–IV). Transthoracic echocardiography (TTE) demonstrated a dilated left ventricle with severe systolic dysfunction (biplane ejection fraction 27%) and a heavily calcified tricuspid aortic valve with severe low-flow, low-gradient aortic stenosis (aortic valve area 0.8 cm^2^; mean gradient 21 mmHg). The Tendyne prosthesis was well seated with a mean transmitral gradient of 3 mmHg and trace regurgitation. Multidetector computed tomography demonstrated an aortic annular area of 406 mm^2^, perimeter 72.2 mm, with left and right coronary heights of 13.8 and 15.3 mm, respectively. LVOT obstruction risk was assessed by qualitative evaluation of the aortomitral curtain through multiplanar computed tomography reconstructions, confirming preserved spatial clearance between the anterior Tendyne frame and aortic annulus suggesting a low risk. The iliofemoral vessels were of adequate caliber, without significant tortuosity or calcification, confirming suitability for transfemoral TAVI.

Under general anesthesia, balloon aortic valvuloplasty was performed using a 22 mm True balloon (Video 1). Subsequently, a 27-mm Navitor (Abbott, London, UK) TAVI was advanced via the transfemoral approach and deployed under rapid ventricular pacing (120/min) (Video 2). A self-expanding Navitor platform was selected to allow controlled, gradual deployment and minimize the risk of interaction between a balloon-expandable system and the adjacent Tendyne frame. The valve expanded symmetrically with optimal positioning and satisfactory hemodynamic performance, with a postimplant mean transvalvular gradient of 6 mmHg and mild anterior paravalvular regurgitation on TTE. The Tendyne mitral prosthesis remained well seated with stable transmitral gradients and no evidence of LVOT obstruction. The patient had an excellent recovery and was discharged 24 hours postprocedure. At 30 days, the patient remained clinically well, with stable valve hemodynamics, preserved Tendyne function, and no LVOT obstruction on TTE.

To our knowledge, this represents the first reported case of TAVI for native aortic stenosis in a patient with a pre-existing TMVR (Figure 1). Previous publications have described the reverse sequence (TAVI followed by TMVR),^2^ as well as valve-in-Tendyne procedures^3^; however, the TAVI performed after prior Tendyne implantation has not been previously reported. This case highlights the importance of preprocedural planning to define the spatial relationship between the Tendyne frame and aortic annulus ensuring safe device interaction with an altered left ventricular geometry. The procedure demonstrates the technical feasibility and favorable hemodynamic performance of TAVI following prior Tendyne TMVR, thereby extending the therapeutic possibilities for high-risk patients with complex multivalvular disease.

**Figure 1:**
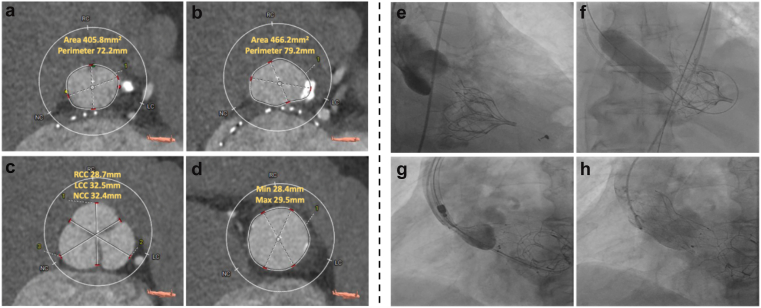
**Preprocedural CT assessment and procedural fluoroscopy during TAVI. (a–d)** Preprocedural multidetector computed tomography (CT) analysis: **(a)** aortic annulus measurements (area = 405.8 mm^2^; perimeter = 72.2 mm); **(b)** left ventricular outflow tract (LVOT) measurements (area = 466.2 mm^2^; perimeter = 79.2 mm); **(c)** sinus of Valsalva dimensions (right coronary cusp [RCC] = 28.7 mm, left coronary cusp [LCC] = 32.5 mm, noncoronary cusp [NCC] = 32.4 mm); and **(d)** sinotubular junction measurements (minimum diameter = 28.4 mm, maximum diameter = 29.5 mm). Multiplanar reconstructions were additionally reviewed to demonstrate preserved clearance between the Tendyne frame and aortic annulus, supporting a low risk of LVOT obstruction. **(e–h)** Fluoroscopic images during transcatheter aortic valve replacement (TAVR): **(e)** baseline aortogram; **(f)** balloon aortic valvuloplasty with a 22 mm True balloon; **(g)** partial deployment of a 27 mm Navitor valve; and **(h)** final fully deployed Navitor 27 mm valve in optimal position. Abbreviation: TAVI, transcatheter aortic valve implantation.

## Consent Statement

Written informed consent was obtained from the patient. All reasonable efforts were made to ensure anonymity and that no identifying information would be disclosed.

## Funding

This work was supported by the British Heart Foundation [grant number FS/CRTF/25/24841]

## Disclosure Statement

Rajan Rehan is supported by an RACP Research Establishment Fellowship.

The other authors had no conflicts to declare.

## Supplementary Data

Video 1 Balloon aortic valvuloplasty with a 22 mm True balloon.

Video 2 Final deployment of Navitor 27 mm valve in optimal position.
